# Synthetic biomedical data generation in support of *In Silico* Clinical Trials

**DOI:** 10.3389/fdata.2023.1085571

**Published:** 2023-08-15

**Authors:** Alena Simalatsar

**Affiliations:** ^1^Institute of Systems Engineering, University of Applied Sciences and Arts - Western Switzerland, Sion, Switzerland; ^2^SENSE - Innovation and Research Center, Sion, Switzerland; ^3^SENSE - Innovation and Research Center, Lausanne, Switzerland

**Keywords:** *In Silico* Clinical Trials, Computer-Aided Clinical Trials, Virtual Clinical Trials, virtual cohort of patients, medical devices, synthetic data, biomedical data, data generation

## Abstract

Living in the era of Big Data, one may advocate that the additional synthetic generation of data is redundant. However, to be able to truly say whether it is valid or not, one needs to focus more on the meaning and quality of data than on the quantity. In some domains, such as biomedical and translational sciences, data privacy still holds a higher importance than data sharing. This by default limits access to valuable research data. Intensive discussion, agreements, and conventions among different medical research players, as well as effective techniques and regulations for data anonymization, already made a big step toward simplification of data sharing. However, the situation with the availability of data about rare diseases or outcomes of novel treatments still requires costly and risky clinical trials and, thus, would greatly benefit from smart data generation. Clinical trials and tests on animals initiate a cyclic procedure that may involve multiple redesigns and retesting, which typically takes two or three years for medical devices and up to eight years for novel medicines, and costs between 10 and 20 million euros. The US Food and Drug Administration (FDA) acknowledges that for many novel devices, practical limitations require alternative approaches, such as computer modeling and engineering tests, to conduct large, randomized studies. In this article, we give an overview of global initiatives advocating for computer simulations in support of the 3R principles (Replacement, Reduction, and Refinement) in humane experimentation. We also present several research works that have developed methodologies of smart and comprehensive generation of synthetic biomedical data, such as virtual cohorts of patients, in support of *In Silico* Clinical Trials (ISCT) and discuss their common ground.

## 1. Introduction

Due to the variety of regulations tightly coupled with country-specific legislation, the process of bringing novel medical devices to market presents significant challenges from both engineering and legal perspectives. However, there are constant attempts to harmonize compliance standards with the aim of facilitating market access for medical devices at the international level while preserving consumer safety. Thanks to this harmonization, some of the essential steps of the authorization procedure are common across many countries. For example, medical devices are generally classified into low-, medium-, or high-risk classes, which define the authorization pathway to be taken by small or medium enterprises. The regulatory pathway required for medium-to-high and high-risk medical devices demands scientific evidence of their safety and efficacy, which is usually built within Clinical Trials (CTs).

CTs are a cyclic procedure that may involve multiple redesigns and retesting. Typically, CTs for medical devices take 2 or 3 years and cost between 10 and 20 million € (VanNorman, 2016). The global medical device market is estimated to be $495.46 billion in 2022 and is expected to grow up to $718.92 billion by 2029 at a CAGR of 5.5% in forecast period^1^, where Western EU and North US markets account for one third each of the global market. The Swiss medical device market is smaller and was valued at $23 billion in 2021. However, Switzerland is also less populated than its neighboring Western EU countries with only ~9 million residents. Nevertheless, Swiss medical device manufacturers are competitive in the global marketplace and almost three times more medical devices are exported than imported.^2^ According to Akpinar (2018) clinical trials for medical devices contribute to 4% of the device's total cost. This way we can estimate that about $20 billion worldwide is spent only for clinical trials, while this estimate does not include salaries and person-months spent for this activity.

In the Swissmedic annual report for 2017 (Swissmedic, 2017), it was stated that the Agency Council is currently working on developing strategies to deal with “the rapid pace of transformation in sciences and technologies" in the coming years. At the same time, the US Food and Drug Administration (FDA) acknowledges (Owen and Jeffrey, 2017) that practical limitations for many devices require alternative approaches. They believe that computer modeling and engineering tests will help to conduct large randomized studies for high-risk medical devices, such as closed-loop drug delivery pumps or pacemakers. Therefore, in 2020, the FDA created a Digital Health Center of Excellence to align and coordinate digital health work across the FDA and keep pace with rapidly evolving digital health research and innovation. The straightforward idea behind the use of computer modeling in place of randomized studies would be to replace real individuals participating in CTs with a virtual cohort of patients and perform *in-silico* CTs (ISCT). However, even though this idea has been around for many years, the full-scale adoption of ISCT is far from reality due to the high complexity of regulatory procedures on one side and the high level of sophistication of computational models in this research domain on the other side.

Within this paper, we address this two-fold problem and show how the generation of synthetic Big Data can be used in place of CTs. First, we present a coherent view of the regulatory pathways of medical devices to market. Further, we give an overview of several medical domains where computational approaches involving the generation of synthetic biomedical data are currently being applied or have a high potential to be applied in the near future. While the core of our paper is a detailed discussion of ISCT adoption for three examples of high-risk medical devices safety and performance testing.

The paper is organized as follows. We begin Section 2 with a description of general initiatives advocating for the use of ISCT with the aim to apply the Replace, Reduce, and Refine (3Rs) principles in humane and animal experimentation. In Section 2.1, we provide a generalized and simplified overview of the medical device regulatory pathways to market. Further, we give a definition of Medical Cyber-Physical Systems (MCPS) in Section 2.2 followed by Section 2.3 where we focus on essential elements of ISCT for high-risk Physiological Closed-Loop Control (PCLC) medical devices, a subclass of MCPS. In Section 3 we discuss three examples of adoption of ISCT for devices testing at different stages of their life cycle, while the discussion of their common ground is presented in Section 4. The conclusion is drawn in Section 5.

## 2. Data generation to create virtual patients for clinical trials

The idea of using patient-specific computational models in the development, evaluation, and testing of drugs, medical devices, or even medical interventions has been around for quite a long time in different research domains. For example, in the domain of medical imaging, the first virtual patient models, such as 3D graphical models of the brain, were introduced in the late 1990s (Collins et al., 1998). There also exist various Computational Human Phantoms (CHPs) with virtual organ models, as well as techniques and semi-automated segmentation tools allowing fast development of CHPs (Kainz et al., 2019). All of these synthetic models have been created to replace often expensive or hard-to-realize MRI and radiology experiments needed for testing novel diagnostic devices, software, or procedures involving medical imaging. In the domain of medical imaging, the use of graphical phantoms for regulatory evaluation is referred to as “Virtual Clinical Trials.”

In 2007, the first idea of creating a European non-profit organization focused on the development of Virtual Physiological Humans (VPH) was published by the consortium of the STEP project in a consensus document entitled “Seeding the EuroPhysiome: A Roadmap to the Virtual Physiological Human” (STEP, 2007). In 2009, it was proposed again and, after the great success of a public petition organized to verify the community's support for the creation of a VPH institute, it was officially incorporated in 2011 in Belgium. The creation of the VPH institute, which unified research from different biomedical modeling domains, led to an even larger initiative: the creation of the Avicenna Alliance.^3^

In 2013, the European Commission funded the Avicenna Action, whose purpose was to elaborate a general roadmap for using physiology-based human models for the medical product regulatory process with the goal of Reducing, Refining, or Replacing the number of animals and humans used in experimental CTs. This action initially brought together around 200 experts represented by researchers, biomedical industries, clinical trials service providers, and regulators and almost tripled this number within two years. As a result of this action, the non-profit Avicenna Alliance was created in 2016 with the mission of large-scale adoption of computer modeling and simulation in place of human and animal experiments, calling such an approach “*In Silico* Clinical Trial” or ISCT. Multiple positioning papers have been published since then, among which we would like to mention one summarizing the results of the Avicenna Action (Viceconti et al., 2016), and two more (Pappalardo et al., 2018, 2022) that continue to communicate the idea and challenges of ISCT within the research community. These publications include a very large set of references to works where computational models in biomedical research could or were used to support the ISCT approach.

Within the same decade, from 2010 to 2020, several groups of computer scientists outside Europe presented their approaches for testing the performance and safety of several medical devices (Kovatchev et al., 2009; Man et al., 2014; Ivanov et al., 2015; Jiang et al., 2016), including drug delivery devices, such as the Generic Infusion Pump (GIP) initiative (Kim et al., 2011; Murugesan et al., 2017) involving multiple US players, the artificial pancreas (Kovatchev et al., 2009; Man et al., 2014), and the closed-loop anesthesia delivery pump (Simalatsar et al., 2018a,c), often referring to the approach of generating a virtual cohort of patients using specific computational models as “Computer-Aided Clinical Trials” or CACT.

Three of the terms, Virtual Clinical Trials, CACT, and ISCT, have a common meaning behind them that incorporates the idea of using patient-specific computational models to generate a virtual cohort of patients in the development or regulatory evaluation phase of medical devices. Respecting the Avicenna action, which has the most ambiguous goal of generalizing the use of computational models in the regulatory pathway, within this paper, we will stick to their term—“*In Silico* Clinical Trials (ISCT).”

The greatest advantages of using ISCT are obvious:

- It allows for the generation of a larger virtual cohort of patients, far exceeding the number of patients that can be involved in real clinical studies.
- The results are easily reproducible at no additional cost.

The idea of generating a synthetic cohort of patients has been around for many years, and numerous papers have been published describing the use of computational models for the discovery, design, development, and testing of medical devices, as well as for statistical testing of endpoints for market approval (Kovatchev et al., 2009; Man et al., 2014; Viceconti et al., 2016; Pappalardo et al., 2018, 2022). However, the full-scale adoption of ISCT is still far from reality. Several factors are preventing the use of ISCT in place of CTs. Firstly, the regulatory procedures are challenging for representatives of the research and development community to comprehend. Researchers are often focused on non-profit goals, with mostly social impact, and in the best-case scenario, potential market size for their innovation, neglecting the endpoints that must be proven in CTs for successful market entry. Furthermore, biomedical computational models are often complex and challenging for regulators to understand. It is challenging to imagine how these models could be used to prove the safety, robustness, security, and efficacy of novel medical devices that are increasingly based on software technologies.

To address these challenges, several initiatives have recently emerged. Among them, we would like to mention first and foremost the Digital Health Center of Excellence^4^, which is a part of the US FDA. It was launched in September 2020 in order to keep pace with the rapid evolution of information technologies in the biomedical domain. Among other things, they provide scientific expertise across the FDA and transparently share resources for developers, thus providing a comprehensive approach to the adoption of digital health technology for both internal and external FDA stakeholders.

We would also like to mention the novel Biomedical Data Science Center (BDSC)^5^, which was created in Lausanne, Switzerland, in 2021 as a joint effort of the University of Lausanne and CHUV, the largest public hospital in Lausanne. The main objective of the center is to promote the use of biomedical big data to enable personalized medicine by generating synthetic medical data.

In full support of all the above-mentioned activities, in this paper we will first attempt to simplify the understanding of regulatory procedures for the research community, providing the level of detail that should be enough to raise awareness that even at the research stage of product development, scientists may already envision potential endpoints of clinical trials.

### 2.1. Medical device pathway to market

One of the main and oldest globally recognized agencies responsible for the authorization of novel medical devices entering the market is the United States Food and Drug Administration (FDA or USFDA)^6^ federal agency. In the European Union (EU), this function is taken on by the European Commission and European Medicines Agency (EMA).^7^ Switzerland, a country with a large medical devices market geographically located in Europe, but not a member of the EU, has the Swiss Agency for Therapeutic Products (Swissmedic)^8^ that plays the role of surveillance authority for medicines and medical devices.

Due to the variety of regulations tightly coupled with country-specific legislation, which are often changed according to novel economic and political agreements, the process of bringing a novel medical device to market presents significant challenges from both engineering and legal perspectives. Already, the definition of medical devices as well as their classification varies across different countries.

To facilitate market access for medical devices at the international level, the European Commission, EMA, FDA, and Swissmedic work together to share best practices in compliance standards, aiming to reduce duplication of inspections on each other's territory while preserving consumer safety (Castle et al., 2017; EMA, 2022). Such harmonization at the international level requires internal alignment of country legislation regarding medical products, and externally, the signing of bilateral mutual recognition agreements (MRAs) between countries' authorities concerning the conformity assessment of regulated products.

Recently, complete alignment of Swiss medical device legislation according to the EU Medical Devices Regulation (MDR) and *In Vitro* Diagnostic Medical Devices (IVMD) took place on 26 May 2021 and 2022, respectively. However, they failed to sign a bilateral agreement, and therefore, from a legal point of view, Switzerland has been classified as a third country in relation to those regulations, implying the need for manufacturers to mandate an authorized representative in another respective EU member state.

Nonetheless, continuous efforts toward harmonization of compliance standards enable us to view the various agencies responsible for authorizing new medical devices as part of a global entity, where the best practices of one agency can be adopted by another eventually. Therefore, in this section, we will provide a generalized overview of the medical device authorization procedure, based on commonalities among most agencies, without claiming complete accuracy in legal details. We will also interpret the regulations' statements regarding new approaches aimed at improving consumer safety, and focus on the 3R principles in human and animal experimentation, which are shared by all agencies.

Let us consider any device intended to be used for medical purposes as a medical device. The pathway to the market of any medical device depends on its classification. In the US, medical devices are classified into three groups: low (Class I), medium (Class II), and high-risk (Class III) devices. In Europe, medical devices are classified into four classes: low (Class I), medium (Class IIa), medium-to-high (Class IIb), and high-risk (Class III) devices. Japan also has four classes ranging from insignificant to high-risk that could cause life-threatening harm, while the rest of the world's classifications are usually similar to those three. The class assignment to a particular medical device is based on estimations of the potential severity, reversibility/non-reversibility, and duration of harm that malfunctioning of the device can cause.

The regulatory control procedures can be generally divided into three types:

1. *General control*, which includes mostly legal formalities regarding product registration. This is applied to low-risk Class I medical devices.
2. *Special control*, which may include special labeling requirements, mandatory performance standards, and post-market surveillance. This procedure is usually applied to medium-risk Class II medical devices.
3. *Premarket approval*, a procedure based on scientific review with the aim to ensure the device's safety and effectiveness, applied to medium-to-high and high-risk Class IIb and Class III medical devices.

The scientific evidence of the safety and efficacy of a novel medical device, or drug, is investigated during premarket approval. Clinical trials are designed to test hypotheses and rigorously monitor and assess trial results on humans. Thus, they must be approved by a supervising ethics committee of relevant countries before permission to run the trial is granted. The goal of clinical trials is not only to ensure scientific validity but also reproducibility of the results. Therefore, the trials can be seen as an application of a scientific approach, the experimental step especially.

Most commonly, CTs are run on drugs, medical devices, and medical procedures and follow the roadmap of three phases: *phase I*—testing with a small group of people, *phase II*—testing on a large group of people, and *phase III*—post-marketing studies, representing surveillance during the lifetime of use/application of a drug, medical device, or procedure (FDA, 2018). However, while the steps taken to go through CT phases are well-defined and clear for the pharmaceutical domain, they are not standardized for medical devices and are case-by-case based. This opens the doors to the use of alternative scientific approaches, such as ISCT, involving computer modeling and engineering tests to help perform randomized studies.

On average, the whole cycle of premarket phases of CTs for drugs takes eight years, while for medical devices it can be significantly shorter, mostly due to the fact that novel medical devices are mostly incremental developments and innovation often only affects a few elements. In recent years, the idea of drug repurposing has gained a lot of attention (Roy et al., 2021), since it can drastically reduce the costs of drug discovery by taking an incremental or pivotal path in CTs similar to medical devices.

### 2.2. Medical cyber-physical systems

**Figure 1:**
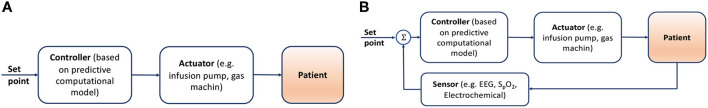
**(A)** Open-loop controller, where the controller makes decisions based on some average model, not allowing personalization. **(B)** Closed-loop controller, incorporates real-time measurements of the patient's state into the decision-making, allowing personalization.

Medical devices from medium-to-high and high-risk groups are often represented by complex Cyber-Physical Systems (CPS), increasingly based on software technologies, that can be classified as Medical Cyber-Physical Systems (MCPS). MCPS is a distinct class of CPS (Lee et al., 2012) with a primary focus on the medical domain, which combines embedded software, control devices, and the complex physiological dynamics of patients. The “physical" part refers to humans or individual organs under treatment, the physiological state of which is defined by the value(s) of physiological variables. The “cyber" part, in turn, is represented by software, such as digital target therapy controllers, decision-making algorithms, medical alarms, or even computational platforms for medical device interoperability. Within this paper, we focus on MCPS with digital target therapy controllers, which are algorithms that make decisions about the treatment of the target state/disease based either on a predictive computational model (i.e., open-loop controller, see Figure 1A), or on feedbacks–the measured physiological values–themselves or mixed with computational models (i.e., closed-loop controller, see Figure 1B). The principal difference between the two systems is the presence or absence of the feedback loop.

Both approaches include the controller component, separated from the actuator since these two components represent different functionality. The controller makes a decision about the treatment that needs to be provided, and the actuator ensures that this treatment is possible within the given constraints of the system. For example, in a drug delivery system, the controller will make a decision about the delivery rate of intravenously (IV) injected medication. The actuator will adapt the speed and verify if such a speed is within the physiological limits of IV injection, and raise an alarm if the syringe with the medication is about to be empty and needs to be refilled.

In the open-loop cyber-physical systems (see Figure 1A) the controller component makes a decision about the treatment based only on an average computational model. Let us consider an infusion pump, a medical device that delivers fluids, such as nutrients and medications, into a patient's body in controlled amounts. The class of computational models used in infusion pump controllers are predictive models built based on medical evidence, such that they are able to predict the outcome of the treatment for the whole spectrum of possible treatments. Therefore, the task of the controller here is to choose the desirable outcome and send a command to the actuator. The quality of the decision depends only on the quality of the average predictive model, since no actual feedback on the effect of the treatment performed by the actuator on the patient is available. Open-loop controllers can be a good fit for systems performing low-risk treatments, such as delivering medical substances with a large therapeutic range, e.g., vitamin C or paracetamol, where the use of only an average computational model is safe and effective.

For medical substances with a narrow therapeutic range, such as anesthetics or cancer drugs, using only the average population model can result in imprecise treatment for a large group of individuals. For example, the widely used Orchestra Base Primea target-controlled infusion (TCI) system of Fresenius for intravenous (IV) anesthesia delivery uses a classic open-loop algorithm (Shafer and Gregg, 1992) based on average population Pharmacokinetic (PK) models for the anesthetic propofol. The inaccuracy of concentration prediction by the algorithm can reach up to 100% for certain individuals (Eleveld et al., 2014), a fact that is well illustrated in Figure 6.

Thus, if one aims to build a device that provides a certain level of treatment personalisation, it requires the introduction of a feedback loop, i.e., closing the control loop of the device, which falls into the class of physiological closed-loop control (PCLC) systems (see Figure 1B). PCLC is a medical device that, in addition to the controller and actuator, includes sensors that can measure physiological variables. The controller must include a feedback loop mechanism, such as an algorithm, which can either simply manipulate the physiological variables through direct activation of therapy or by personalizing the computational model in use.

Considering the variability of medical devices capable of measuring specific physiological parameters and making decisions about patient treatment based on their values, it is hard to have an effective benefit-risk evaluation of such complex medical systems. One of the main reasons for that is the absence of a systematic classification of such critical care devices as well as a framework for their level of automation.

To facilitate the development of autonomous medical devices involved in patient treatment, in 2015 FDA organized a workshop to discuss the main problems and tendencies in the development of novel PCLC devices (Parvinian et al., 2018). Among the target devices classified as PCLC, the FDA listed:

1. Automatic anesthesia delivery;
2. Fluid resuscitation/vasopressor delivery, and
3. Mechanical ventilation.

The FDA also recognizes that since the physical implementation of PCLC devices involves both hardware and software components, it is essential to extend standard safety considerations for medical devices, such as biocompatibility, sterility, and electrical safety, to include those related to

- hardware limitations (e.g., sensors and actuators) and
- software robustness (digital controllers),

as well as stable interoperability among all the aforementioned components. Further classification and specification requirements are necessary to ensure the safe and effective use of these devices.

### 2.3. Generalized classification of PCLC devices

The first two device classes listed by the FDA (Parvinian et al., 2018)—automatic anesthesia and fluid resuscitation/vasopressor delivery—fall into a more general class of closed-loop drug delivery devices that can also include the delivery of analgesics, myorelaxants, chemotherapy during cancer treatment, or even hormone delivery devices. The most vivid example of closed-loop drug delivery is the case of the Artificial Pancreas (AP), which is used to treat diabetes. All of these pharmacological treatments require either continuous injection during a certain period of time with real-time delivery rate adaptation or a series of continuous injections with a fixed rate, while the dose is adapted from one series to another. The difference in approach depends on how fast the drug is being eliminated by the body, with real-time delivery rate adjustment being required for drugs with fast clearance of the medication. The third class, mechanical ventilation devices, may seem to fall into a separate class of medical devices. However, we can view oxygen as a chemical substance the delivery of which is regulated in real-time based on indirect SpO_2_ measurement.

This way, all of the three device classes mentioned above can be classified as non-implantable Substance Delivery Devices (SDDs). Some SDDs are only used for short-term treatment and therefore will remain non-implantable, while the size, safety, connectivity, and usability properties of others will continue to improve. Some devices, such as artificial pancreas, are currently non-implantable or only partially implantable due to the high risks associated with closed-loop delivery and the need to refill reservoirs. However, once these challenges are solved, we may see an implantable version of the AP.

There are also PCLC devices built using hardware and software components that cannot go through the non-implantable phase of tests and adaptations and must be implanted from the first day of their development, such as Implantable Cardio Defibrillators (ICD). ICDs are electronic devices that are usually placed near the heart and connected to it to detect and treat chaotic fibrillation by delivering an electric shock to restore normal heart rhythm. The increased risks associated with the necessity of surgical operation for device implantation and the absence of a possibility to quickly extract the device and replace it with an alternative efficient treatment in case of malfunctioning raise the demand for device safety and efficacy compared to non-implantable PCLC devices.

## 3. *In-Silico* Clinical Trials case studies

In this section, we will present case studies to illustrate how computational models can generate virtual cohorts of patients and be used for testing medical devices, with a focus on ISCT for evaluating the safety and efficacy of novel PCLC medical devices. We will start with a series of research works on the application of ISCT for ICDs. In this case, ISCT was applied to previously approved devices, allowing for a comparison of efficacy with regular CTs, as well as proposing a mixed approach where prior information about clinical trials was included in the ISCT process. Our second example is an ISCT performed for a closed-loop anesthesia delivery device still in the development stage, where some components were not fully available. Finally, we will present a famous example of a T1DM simulator for artificial pancreas ISCT, which represents an example of the early adoption of a medical device based solely on ISCT simulations.

### 3.1. Implantable cardio defibrillators

According to clinical trials conducted from 2005 to 2010, it was found that ICDs had a high rate of inappropriate therapies (Gold et al., 2012). These electric shocks were delivered in cases where the arrhythmia was not critical and therapy was not necessary, leading to increased patient stress and morbidity. Organizing clinical trials for an implantable device is a complex process that carries significant risks for the intervention group, i.e., patients who receive treatment with an untested device. Additionally, since there are different types of arrhythmias, it is challenging to have a large cohort of patients with a statistical distribution that corresponds to the actual statistical accuracy of various types of arrhythmias.

**Figure 2:**
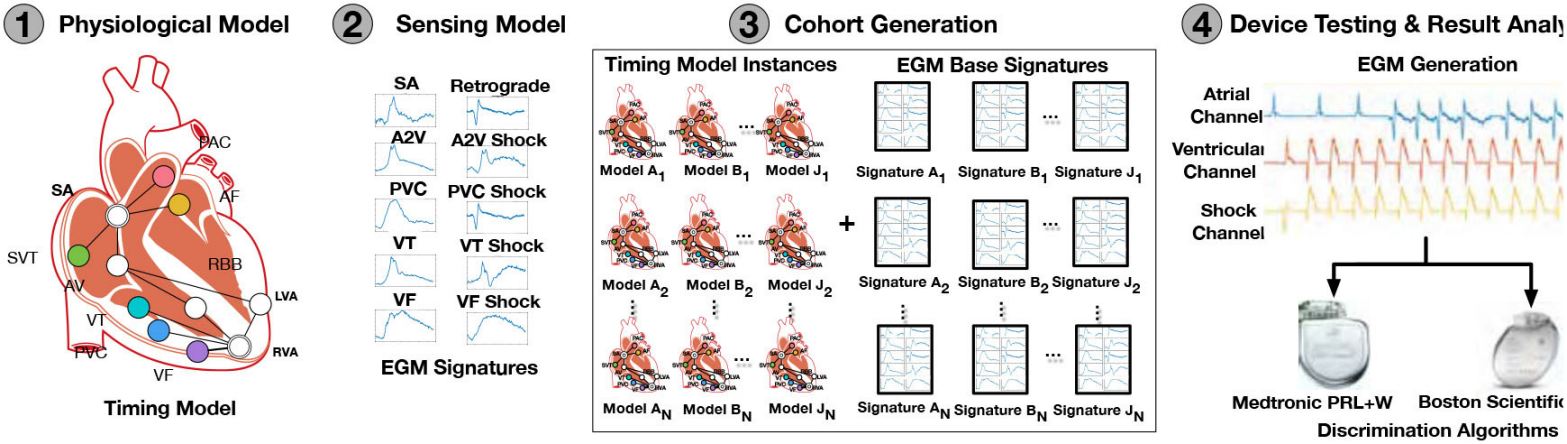
Overview of *in-silico* trials (Jiang et al., 2016).

In their work (Jiang et al., 2016), the authors proposed an approach for *in-silico* testing of such devices, which can be used for early, affordable, and reproducible evaluations, allowing for testing of assumptions made in clinical trials. The schematic overview of this *in-silico* trial is presented in Figure 2 and is composed of:

1. *A physiological model*. The model is based on Electrophysiology (EP) studies. The topology of the electrical conduction system of the heart is represented by the nodes and paths of cooperating automata that model the timing of generation and blocking of electrical events. Thus, tachycardia is modeled as high-frequency events generated by specific nodes.
2. *A sensor model*. To detect the event of tachycardia, the classification algorithm of the ICD uses the timing and morphology of the electrical activity of the signal slice under evaluation. To generate realistic electrogram (EGM) signals, the sensor model selects a signal morphology from a set of 10 available templates corresponding to one of 10 different activation paths overlaid with the timing sequence of electrical events.
3. *Cohort of patients*. A large (>11,000) virtual cohort of patients was generated, covering 19 common arrhythmic conditions as a combination of timing and morphology.
4. *Device testing*. Two algorithms of ICD devices discriminating fatal Ventricular Tachy-arrhythmias (VT) and non-fatal Supra-Ventricular Tachy-arrhythmias (SVT) were tested in terms of evaluating the rate of inappropriate therapies applied, which grossly confirmed the results of the clinical trials performed for these devices earlier.

In addition to the classical advantages of ISCT application in this case, ISCT also allowed for sensitivity and specificity analysis of algorithms. This analysis determined which types of arrhythmia caused the most false-positive classifications, leading to inappropriate therapies. As a result, possible improvements for the algorithms were suggested. For example, it also allowed the study of the effect of parameter values that can be tuned before device implantation on the discrimination capabilities of the algorithm. Such analysis would have been impossible within regular CTs.

The limitation of the study presented above lies in the fact that the generated cohort of patients was homogeneous with respect to different heart conditions. In reality, some types of heart arrhythmia appear more frequently than others. Therefore, in the follow-up work (Abbas et al., 2018), the authors incorporated prior knowledge from real patients involved in real CTs about the distribution of various arrhythmia types, as well as other key parameters captured by the computational model, following a Bayesian approach. A similar Bayesian approach for integrative CTs using both virtual and real patients was applied in a successful mock submission to the FDA describing CTs on fatigue fracture in a hypothetical next-generation ICD lead (Haddad et al., 2017).

In the next paper of this series (Jang et al., 2019), the authors presented a robustness evaluation statistical framework that allows for the evaluation of how the outcome of CTs would change with respect to how many patients with certain types of arrhythmia participated in the studies. This answered questions about the necessary composition of the potential real cohort of patients to be recruited.

### 3.2. Feedback loop control for general anesthesia

**Figure 3:**
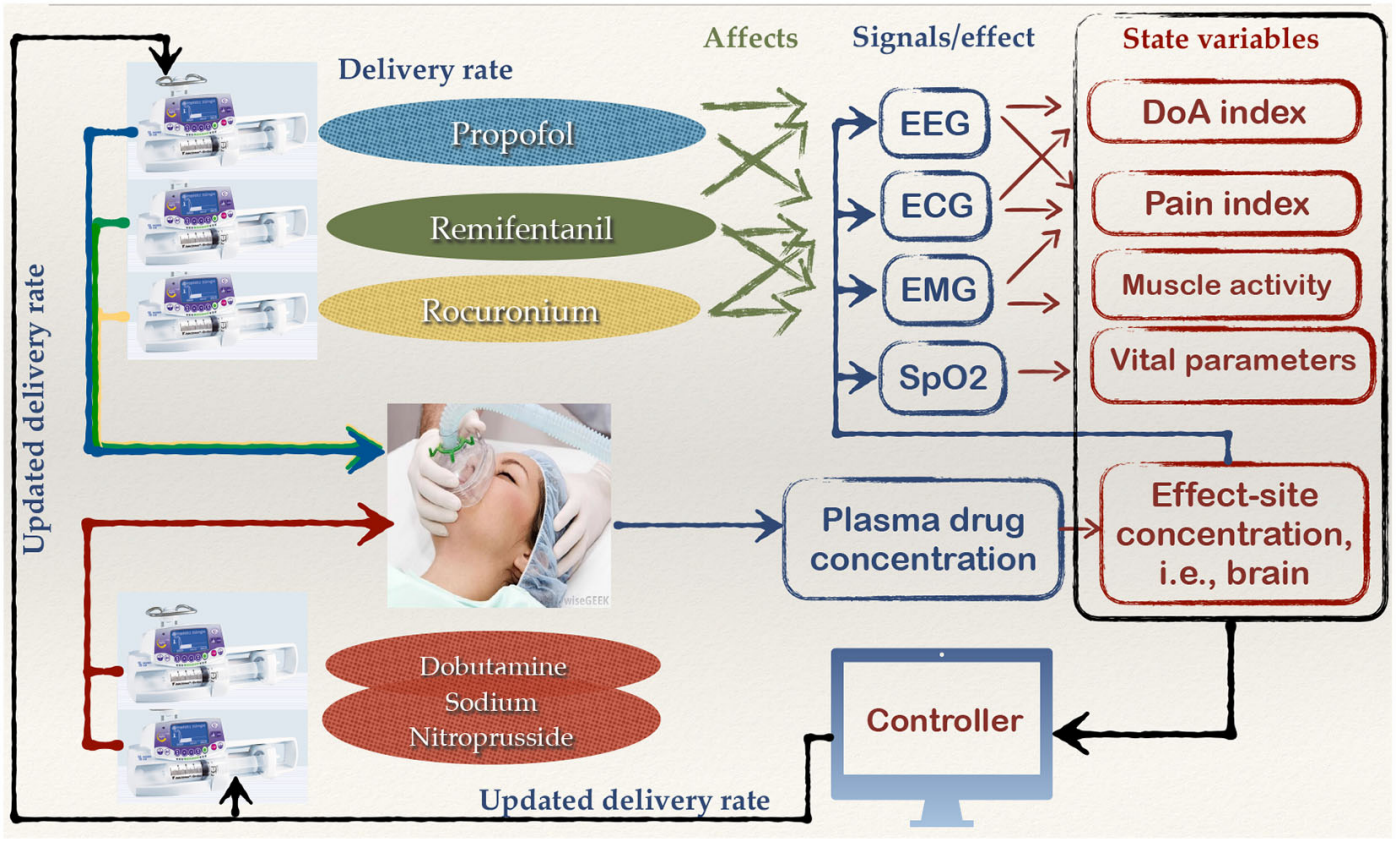
Global view of the components and signals that could potentially help to close the loop for a general anesthesia delivery system.

The closed-loop delivery of general anesthesia is a complex example of ISCT performed for a PCLC device (Simalatsar et al., 2016, 2018a,b,c). The global view of closed-loop general anesthesia delivery with all potential feedback loop information is presented in Figure 3.

As we can see from this figure, general anesthesia is a leveraged delivery of anesthetics, such as *propofol*, analgesics, like *remifentanil*, myorelaxants, such as *recuronium*, and medicaments to lower blood pressure, for example, *dobutamine*/*sodium nitroprusside*, each affecting vital signals including Electroencephalogram (EEG), Electrocardiogram (ECG), Electromyogram (EMG), respiratory rate (RR)/oxygen level (SpO_2_), in an interfering manner. The state variables such as Depth of Anesthesia (DoA) index, pain index, muscle activity, or drug concentrations that can be fed to a digital controller can be derived either from one vital signal or from their combination. Full automation of anesthesia delivery would require reliable patient state variables and a digital controller able to personalize the drug delivery rates based on those variables and a physiological model, which is a very complex task. Only partial solutions for safe general anesthesia automation have been presented so far in scientific literature. For example, closed-loop delivery based on DoA indexes and measurements of anesthetic concentration in plasma, as they are the most direct measures of drug-induced DoA.

#### 3.2.1. Closing the loop with DoA index

The early attempts to close the general anesthesia loop were based on the Depth of Anesthesia (DoA) index (Drover et al., 2002; Kreuer et al., 2003), which provides a statistically derived numerical value ranging from 0 (equivalent to EEG silence) to 100 (awake patient). In particular, there was an attempt to build a closed-loop controller based on EEG-based bispectral index (BIS) (Liu et al., 2011). However, it has been recently proven that existing DoA indexes actually provide an unspecific picture of the brain's responses to anesthetic drugs (An et al., 2017). Therefore, the accuracy of such indexes has been debated as they oversimplify the EEG signal. We can see this as an example of unsuccessful market adoption due to unreliable physiological signal processing algorithms.

Moreover, since the algorithms for existing DoA index computation are proprietary, it is not possible to work on their improvement. Therefore, one needs to start from scratch, which drastically slows down the research and innovation cycle. Hence, there has been an initiative to build a graphical tool (Fluck et al., 2021) that enables quick analysis of EEG signals with a comprehensive set of classical signal processing algorithms. The goal is to speed up research and innovation in domains where EEG signal processing plays a central role.

#### 3.2.2. Closing the loop with medicaments' plasma concentration

Another approach to improving the precision of anesthesia delivery is based on realizing a feedback loop with sensors that can provide real-time measurements of the concentration of the anesthetic, such as *propofol*, in body fluids. Anesthetics are hypnotic agents widely used for the induction and maintenance of general anesthesia. They are usually short-acting intravenously administered drugs with a high elimination rate and are subject to large inter- and intra-patient variability. The therapeutic application of anesthetics requires an ensured multi-step gradation of the effect intensity, i.e., DoA, correlated with the plasma drug concentration. This requires not only continuous injection of the drug but also real-time delivery rate adjustment to keep the drug concentration within a therapeutic range corresponding to the chosen target of DoA and to avoid both patient intoxication and awareness.

Today, anesthetics are often injected using target-controlled infusion (TCI) systems, which can adjust the delivery rate using a classic open-loop algorithm (Shafer and Gregg, 1992) to target a fixed level of plasma or effect site drug concentration. The standard TCI systems only support population (average) pharmacokinetic (PK) models of propofol, which were initially represented by those of Marsh et al. (1991) and Schnider et al. (1998, 1999), and only recently by Eleveld et al. (2014)^9^ models. The decision about the target drug concentration is left to the anesthesiologist.

TCI pumps for anesthesia delivery have not been approved for use by FDA as yet and therefore are being widely used only in Europe and Switzerland. At the moment of the FDA's decision sufficient scientific evidence either proving or disproving TCI safety has not yet been available. Therefore, the rejection of the TCI pump by the FDA cannot be evaluated as evidence-based. It is only with the development of PK models based on much larger statistics and providing not only an average population model but also inter- and intra-individuals variability (Eleveld et al., 2014) it became possible to demonstrate that inaccuracy in drug concentration prediction for certain individuals can reach up to 100% if we only account for inter-individual (between-subject) variability, see Figure 6. Even though the potential error looks dramatic, the advantage of using TCI pumps during anesthesia is still important. Thanks to being representatives of open-loop systems, they allow anaesthesiologists to interfere in the delivery process and adjust the target concentration based on patient vital parameters, while still providing a precious level of drug delivery automation.

Let us look at how one can build scientific evidence about a closed-loop drug delivery device, when sensor technology is not mature enough to build an actual system, with an example of a TCI pump for *propofol* delivery. The core element of the approach is the physiological model, that in the domain of drug delivery devices will be represented with a drug-specific PK model.

##### 3.2.2.1. Physiological model

**Figure 4:**
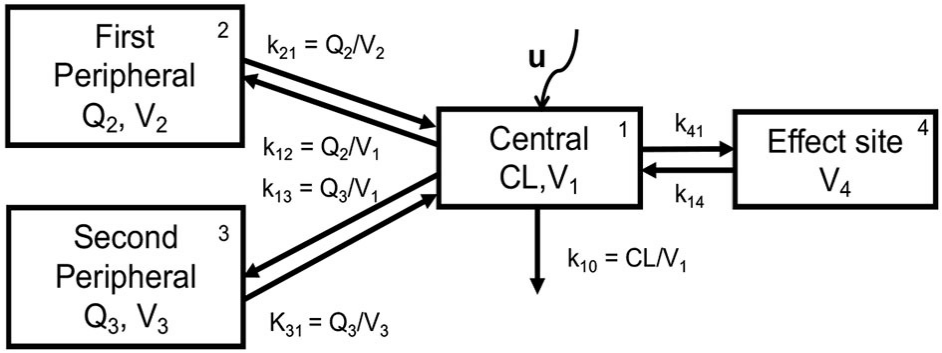
Schematic representation of the 3-compartment PK model extended with the virtual *effect site* compartment (Simalatsar et al., 2018c).

The pharmaceutical industry has a wealth of PK models for different drugs. PK modeling is based on an understanding of the physiology of drug absorption, distribution, metabolism, and elimination by the body, which defines the structure of the model. However, the parameters of the models as well as their inter- and intra-individual variability have a statistical nature and are based on real drug concentration measurements for individuals administered with certain doses of the studied drug, using a Bayesian approach at the model development stage. To give an idea about PK modeling, we will present the PK model of propofol, as shown in Figure 4.

The PK model of *propofol* is usually described by a three-compartment model extended with a fourth virtual compartment representing the effect site, i.e., brain. The system of compartments can be seen as four communicating vessels, where each compartment is characterized by its volume (*V*_*i*_) and clearance (*Q*_*i*_, or CL in the case of the central compartment) that are used to model the drug's distribution and its further release into and by various tissues, regulated by *k*_12_, *k*_21_, *k*_13_, and *k*_31_, which are first-order transfer rate constants from compartment *i* to compartment *j* (see Figure 4). The micro-constants driving central/effect compartments exchange are defined as *k*_41_ = *k*_*e*0_ and *k*_14_ = *k*_*e*0_/10000, where *k*_*e*0_ is the effect site elimination rate constant. The central compartment represents the plasma to which the drug is delivered at a rate of *U* (controlled and changing over time) and is cleared from it with the elimination rate *k*_10_, which is defined as *CL*/*V*_1_.

The system of differential equations—Eqs 1–3,—describes the evolution of drug amounts in the three compartments and concentration in the fourth effect compartment—Eq. 4:


(1) $$\begin{array}{l} \frac{dA_{1}}{dt}=A_{2}k_{21}+A_{3}k_{31}-A_{1}\left(k_{10}+k_{12}+k_{13}\right)+U\left(t\right) \end{array}$$

(2) $$\begin{array}{l} \frac{dA_{2}}{dt}=A_{1}k_{12}-A_{2}k_{21} \end{array}$$

(3) $$\begin{array}{l} \frac{dA_{3}}{dt}=A_{1}k_{13}-A_{3}k_{31} \end{array}$$

(4) $$\begin{array}{l} \frac{dC_{4}}{dt}=\left(C_{1}-C_{4}\right)k_{e0} \end{array}$$

where *A*_*i*_ represents the amount of drug in compartments, while *C*_*i*_ represents the drug concentration. To obtain the concentration in a compartment, the amount must be divided by the corresponding volume of distribution (*C*_*i*_ = *A*_*i*_/*V*_*i*_). The principal difference between different PK models for propofol, e.g., (Marsh et al., 1991; Schnider et al., 1998, 1999; Eleveld et al., 2014), lays in the way the population PK parameters *PAR*_*k*_, i.e., *CL*, *V*_*i*_, and *Q*_*i*_, are computed based on patients' demographic characteristics. This difference leads to different sets of *k*_*ij*_ constants used in differential equations for patients with identical demographic characteristics. In turn, this results in different Concentration-Time (CT) profiles for identical delivery rates *U*. The earlier models (Marsh et al., 1991; Schnider et al., 1998, 1999) provide only equations to compute *PAR*_*k*_, e.g., only population models and inter-individual variability. Only Eleveld's recent model (Eleveld et al., 2014) provides inter- and intra-individual variability.

##### 3.2.2.2. Inter-individual variability

*Inter-individual variability*, accounts for the difference among individuals with identical demographic parameters, and is typically described assuming a log-normal distribution for a given PK parameter:


(5) $$\begin{array}{l} PAR_{k}^{j}=PAR_{k}*e\left(\eta_{k}^{j}\right) \end{array}$$

where $PAR_{k}^{j}$ is one (*k*^*th*^) PK parameters out of *CL*, *V*_*i*_, and *Q*_*i*_, of the *j*^*th*^ individual, *PAR*_*k*_ its average population value and $\eta_{k}^{j}$ is the *k*^*th*^ individual component of the inter-individual random effect, an independent, normally distributed variable with mean 0 and variance $\omega_{k}^{2}$. The inter-individual variability is estimated as ω_*k*_.

##### 3.2.2.3. Intra-individual variability

*Intra-individual variability* accounts for measurement errors in data used during model elaboration, biological fluctuations over the observation period, and inaccuracies inherent to models. The intra-individual variability is more exhaustively described by a combined additive and proportional error model:


(6) $$\begin{array}{l} Y_{n}^{j}=C_{n}^{j}+\sqrt{\left(prop^{2}*{\left(C_{n}^{j}\right)}^{2}+add^{2}\right)}*\epsilon_{n}^{j} \end{array}$$

where $Y_{n}^{j}$ is the plasma concentration measured at step *n* for the *j*^*th*^ individual, $C_{n}^{j}$ is the corresponding predicted concentration, *prop* and *add* are the proportional and additive error terms and $\epsilon_{n}^{j}$ is the *j*^*th*^ component of the random effect, an independent, normally distributed variable with mean 0 and variance 1. A simple additive error model is obtained with *prop* = 0, while a simple proportional error model with *add* = 0.

#### 3.2.3. *In Silico* Clinical Trials for closed-loop propofol delivery

**Figure 5:**
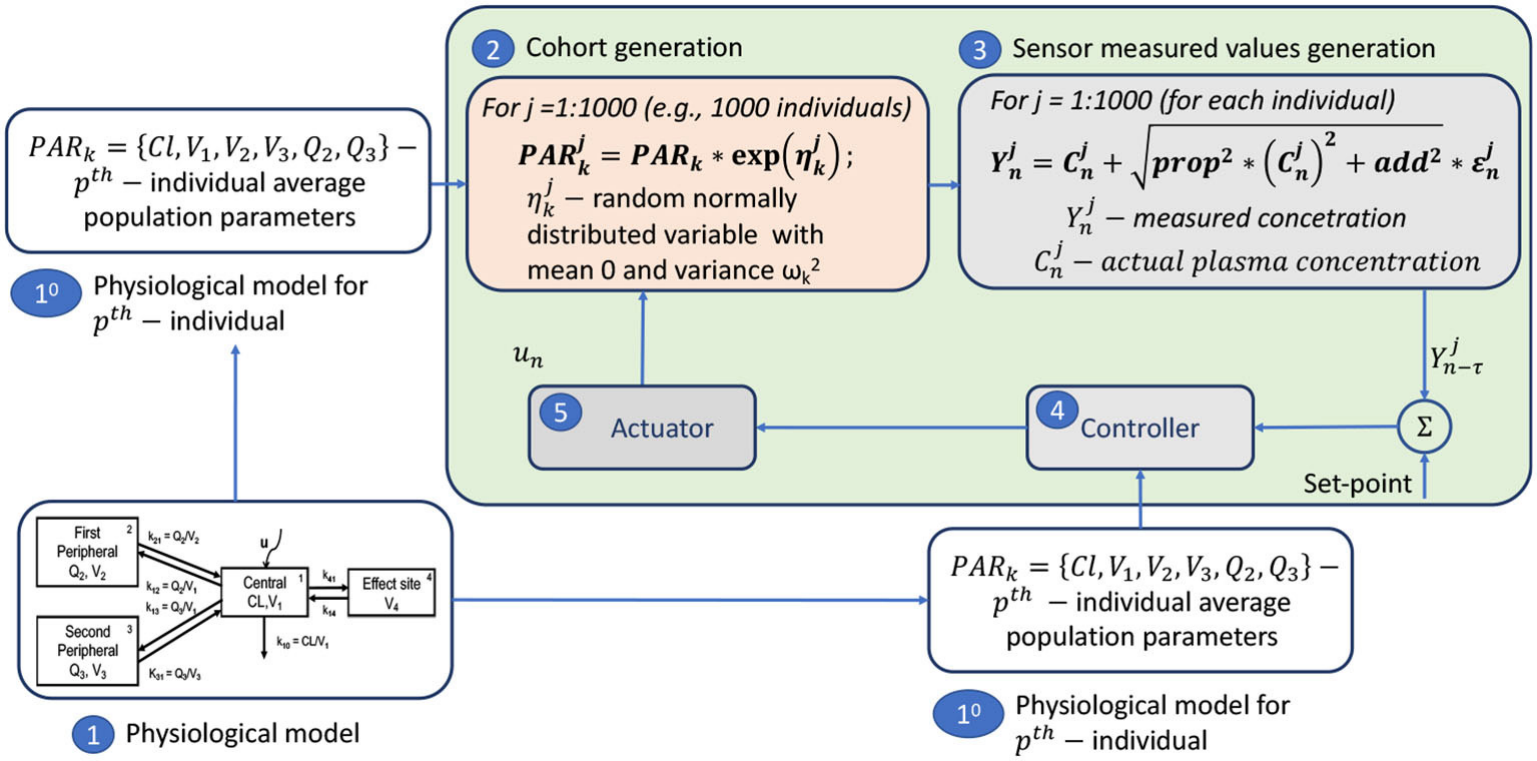
The general flow of ISCT for closed-loop *propofol* TCI pump.

The schematic representation of the ISCT trials performed on the case of closed-loop delivery of anesthetic *propofol* is summarized in Figure 5. This figure represents only a part of the ISCT performed for the case of the system application for individuals with identical demographic characteristics (*p*^*th*^ individuals), as presented in Simalatsar et al. (2018a,b,c). In the figure, we can see that the *physiological model (*1*)* is parameterized with a set of *p*^*th*^ individual average (e.g., population) parameters *PAR*_*k*_ (1^*st*^), thus providing a *p*^*th*^ population PK model used in *cohort generation (*2*)*, as well as used by the *controller (*4*)* for Model-based Predictive Control (MPC).

1. *Cohort generation (*2*):* A virtual cohort of 1,000 36-year-old women, each with a weight of 70 kg and height of 170 cm (denoted as *p*^*th*^ type of individuals) was generated using the inter-patient variability of PK parameters presented above and the values reported in Eleveld et al. (2014). This means that 1,000 new sets of $PAR_{k}^{j}$, corresponding to the *j*^*th*^ individual (*j*∈ [1, 2, ...., 1, 000]), subject to inter-patient variability, were generated. For individuals with different demographic characteristics, the set of population parameters *PAR*_*k*_ will be different and, therefore, will require similar testing with the generation of a new cohort of individuals.
2. *Sensor measured values generation (*3*):* In order to evaluate the performance and robustness of a closed-loop controller (4) under realistic conditions, the intra-individual variability of the Eleveld et al. (2014) model was used to generate statistically sound virtual sensor measurements (Simalatsar et al., 2018a,b,c). Thus, $PAR_{k}^{j}$ was used to compute the plasma concentrations $C_{n}^{j}$ at step *n* for each *j*^*th*^ individual administered with a personalized delivery rate computed by the controller. Those plasma concentrations were further modified following the intra-individual variability described above, to emulate sensor measurement $Y_{n}^{j}$ at step *n* for each *j*^*th*^ individual.
3. *Controller (*4*):* Within this ISCT, the performance and robustness of two MPC controllers adjusting the delivery rate in a personalized manner based on occasional measurements of plasma drug concentration and the *propofol* population PK model were evaluated (Simalatsar et al., 2018a).
4. *Actuator (*5*):* In this closed-loop system, the actuator ensured that the delivery rate set by the controller is actually achievable, taking into account both the limitations of physiology and the delivery mechanism, e.g., the maximum allowed delivery rate.

**Figure 6:**
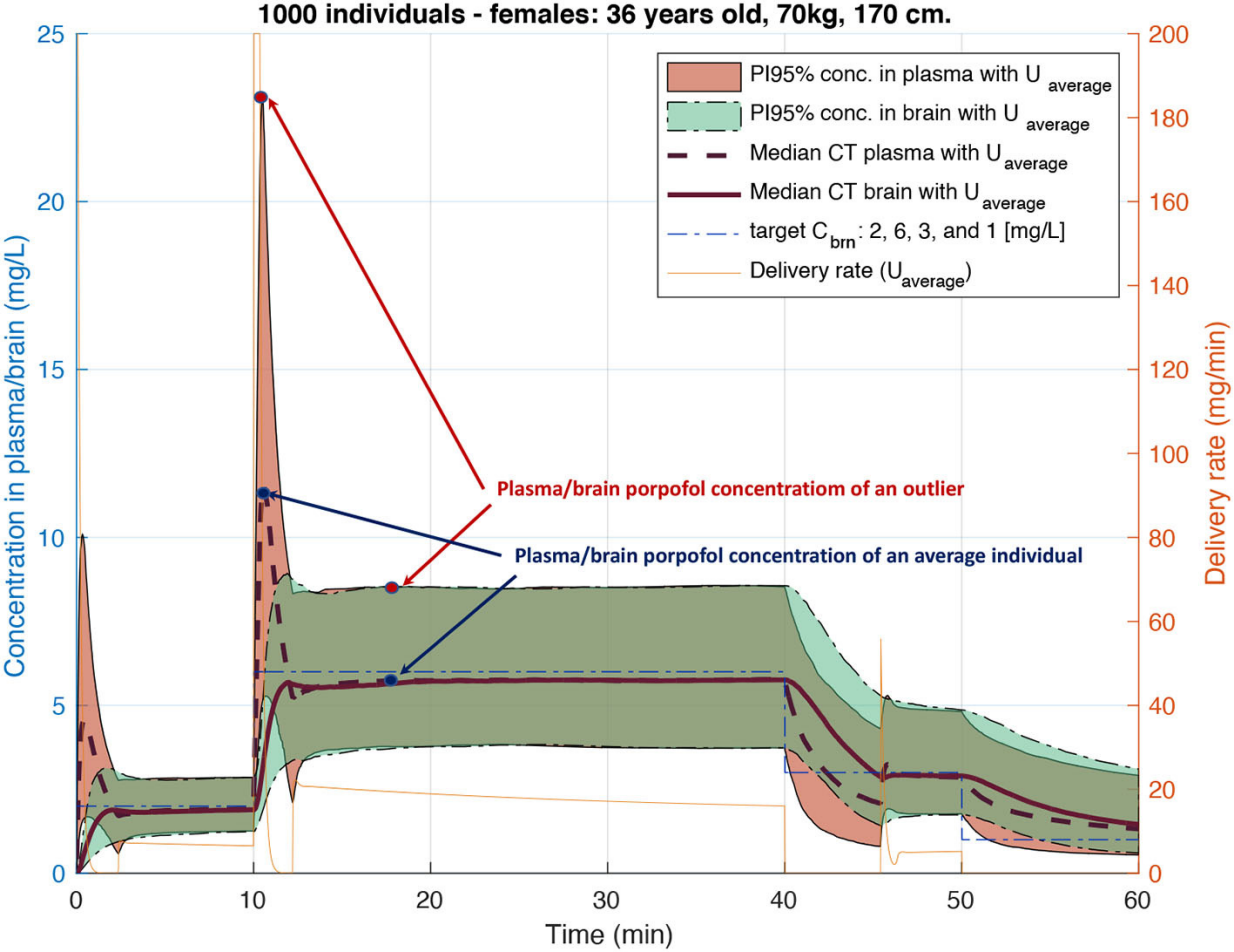
Plasma and brain Concentration-Time (CT) profiles for 1,000 similar individuals with physiological parameters varying following inter-patient variability of Eleveld's model (Eleveld et al., 2014). Each CT distribution is represented with median CT profiles and 95% predictions interval after *propofol* injection with a delivery rate computed using an open-loop algorithm of standard TCI pumps.

*PAR*_*k*_ was used to compute the plasma concentrations $C_{n}^{j}$ at step *n* for each *j*^*th*^ individual, i.e., patient-specific CT profiles, for identical delivery rates computed with the classical algorithm used by the standard TCI pump for the *p*^*th*^ individual. This allowed for the evaluation of potential under- and over-dosing for certain individuals of this virtual cohort, as shown in Figure 6. Such evaluation allowed us to prove the importance of personalization of *propofol* delivery.

The construction of CT profiles for the whole virtual cohort administered with personalized delivery rates allowed performing a robustness analysis of the controller, taking into account several potential sensor technology limitations, thus also imposing requirements for such a technology:

1. *Noise*, defined using proportional (prop) and additive (add) terms of the PK model intra-individual variability.
2. *Measurement period*, the time between two consecutive measurements (*t*_*n*_−*t*_*n*−1_).
3. *Measurement delay*, the time required to perform one physiological measurement ($Y_{n-\tau }^{j}$).

**Figure 7:**
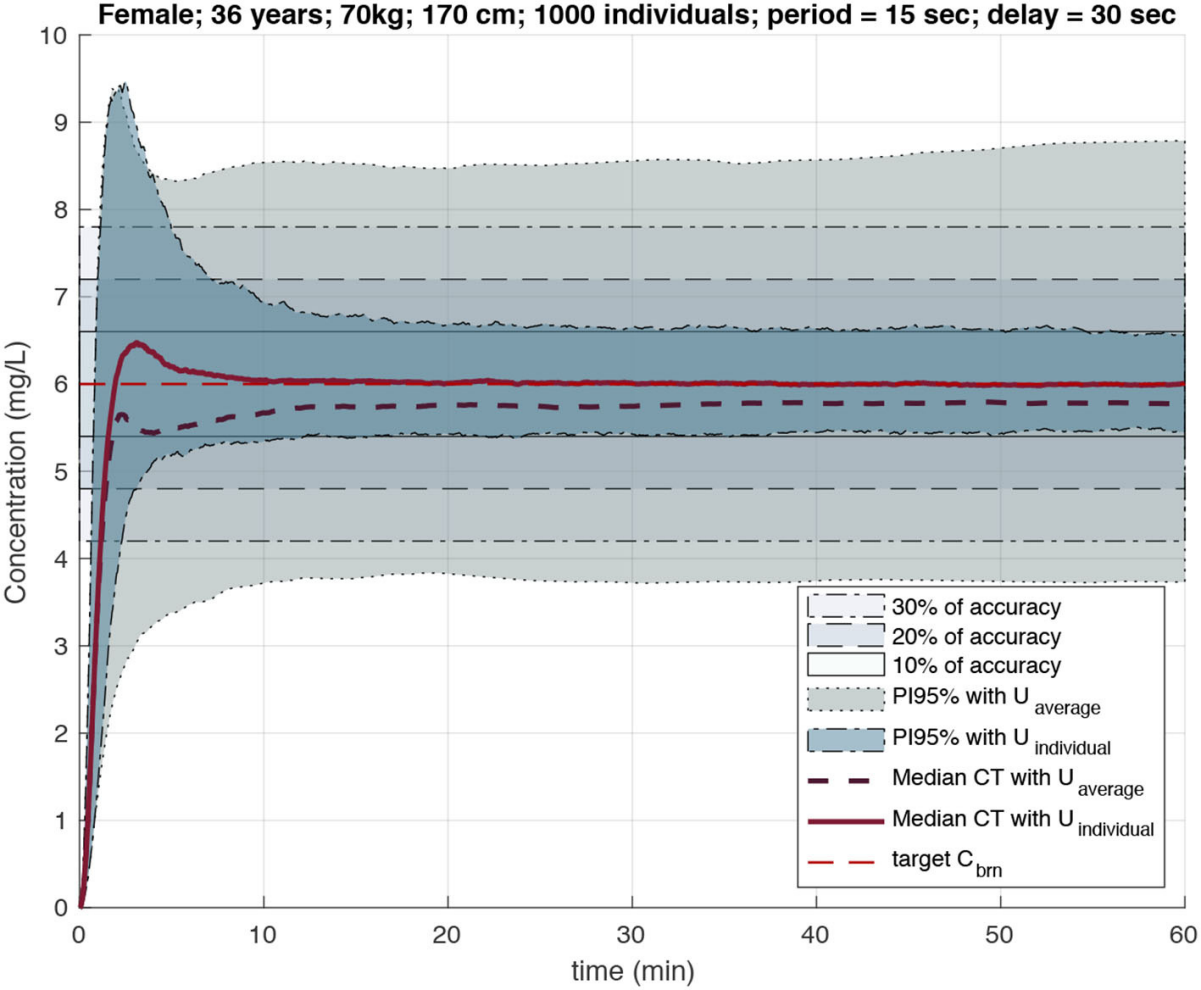
PI95% for effect site and median CT profiles for individuals under the dose regimen computed by standard TCI (larger gray area) and feedback loop algorithm with Bayesian inference (more narrow area around 10% accuracy area). Computations with measurement period of 15 s and delay of 30 s (Simalatsar et al., 2018a).

Their performance (i.e., the precision of target achievement) was compared to that of the standard TCI pump, demonstrating a significant improvement in the precision of target achievement for closed-loop delivery personalized with MPC algorithms and occasional plasma concentration measurements. Specifically, Figure 7 presents a scenario where the target brain concentration is set at 6 mg/L and compares the area of the 95% prediction interval after *propofol* injection with the delivery rate computed using the open-loop algorithm of standard TCI pumps and with the personalized delivery rate when MPC was given one measurement every 30 s, with a value delay of 15 s, and with measurement noise distributed with intra-individual variability for the selected cohort of individuals.

In the example above, we have presented only the use of the PK model, however, the pharmacodynamic (PD) model (Eleveld et al., 2018), which links drug concentration to the actual effect (DoA), has a potential to build and perform ISCT on closed-loop controller based on both DoA index and plasma concentration measurements.

### 3.3. Model predictive control design for artificial pancreas

The classical therapy of type 1 diabetes mellitus (T1DM) consists of a predefined daily schedule of subcutaneous injections of insulin. In 1999, the first commercial Continuous Glucose Monitoring (CGM) system was introduced by MiniMed. Since then, there has been a lot of research done in order to personalize insulin injections with the use of the so-called artificial pancreas, an autonomous system for glycemic control aimed at delivering the amount of insulin based on CGM. This example is similar to the closed-loop *propofol* delivery with TCI pump presented above, where insulin is a medication the delivery rate of which must be controlled based on a sequence of measured glucose levels.

The first version of the type 1 diabetes mellitus (T1DM) simulator of the University of Virginia/Padova was introduced in 2008 (Kovatchev et al., 2009). This simulator included three cohorts of individuals: 100 *in-silico* adults, 100 adolescents, and 100 children, where each subject was modeled as a vector of subject-specific parameters with joint parameter distribution, i.e., inter-patient variability, covering the variability of parameters observed in real-life. For the moment, an artificial pancreas can be considered an exemplary case study of ISCT performed for a PCLC system. This simulator was approved by the FDA as a substitute for preclinical trials for insulin injection pumps driven by closed-loop algorithms and was used in several closed-loop algorithm simulation studies (Lee et al., 2009; Kovatchev et al., 2010; Toffanin et al., 2013) that helped many developers to receive rapid FDA approval of their closed-loop algorithms. An updated version of the T1DM simulator that included a model of counter-regulation and a new description of glucose dynamics in hypoglycemia has been presented in 2013 (Man et al., 2014) and was also approved by the FDA.

In parallel with this research and probably partially based on these results, in November 2012 the FDA issued a guidance for Industry and Food and Drug Administration Staff–The Content of Investigational Device Exemption (IDE) and Premarket Approval (PMA) Applications for Artificial Pancreas Device systems. The interesting part of this guidance is that it suggests that AP systems consist of a number of device components, i.e., glucose monitoring, control algorithm and signal processing, infusion pump, and communication pathways functional components, that all together form a complete system conforming with technological specifications, e.g., the listing of functional components, their interoperability, details of technical features, and clear statement of intended use, defined in the system-level device description. According to the FDA guidance on AP (FDA, 2012), the testing of the control algorithm and signal processing functional component for artificial pancreas solutions should be provided, which currently can be done using the T1DM simulator mentioned above.

## 4. Discussion and common ground

Synthetic big data in the form of virtual cohorts of patients and sensor measurements help to address the problems of safety, efficacy, robustness, and trustworthiness of PCLC at each point of its life cycle, from the early design phase up to CTs. Clearly, the physiological model is a key component used to not only generate a cohort of patients but also to build a sensor model, as described in Sections 3.2.3. It is hard to imagine, but not impossible, that one day we will have a complete physiological model of a human or any other animal, including not only the visual representation but also the mechanical, electrical, and metabolic aspects within one system model. However, for the moment, when we talk about physiological models, we have in mind a domain-specific representation of several aspects of human or animal anatomy, physiology, or, consequently, pathology. Physiological modeling in the pharma domain has been receiving a lot of financial support and is, therefore, represented by a large number of different quite elaborate PK/PD models for different drugs, providing not only average population models but also statistical inter- and intra-patient variabilities. Physiological models outside of the pharma domain are less elaborated (Jiang et al., 2012, 2016; Ivanov et al., 2015).

In the case of PK/PD models, which are built on measurements of real patient responses to treatment, such as plasma concentrations after certain doses, the statistical distribution of patient parameters and measurement imprecision are represented by inter- and intra-individual variabilities, respectively. Other models, such as those used in Jiang et al. (2016) and Haddad et al. (2017), integrate prior knowledge about the statistics/frequency of physiological responses as a second stage of model development using the Bayesian approach. However, they do not differentiate between inter- and intra-patient variability, thus mixing both effects in one variability. The artificial pancreas simulator (Kovatchev et al., 2009) also relies only on inter-individual variability.

To determine the extent to which differentiation between inter- and intra-individual variabilities is needed, one needs to perform sensitivity analysis for each particular case, which will be conditioned by how significant the statistical variability is and how it is used in PCLC design, development, and testing. In the case of general anesthesia, combined inter- and intra-individual variability is large. In addition, the use of only intra-individual variability for emulation of sensor measurements for a fixed individual is more realistic, since inter-individual variability is conditioned by parameters that do not change within one individual in one instant, such as organ sizes, for example.

Let us look at the four key components of PCLC devices shown in Figure 1B. Each of these components may be a potential source of failure, errors, or unpredictable behaviors. Therefore, each of them must address specific requirements and go through validation steps:

### 4.1. Sensors and cohort generation

In all the above-presented examples, sensor models and cohort generation have slightly different meanings. Sensors are the medical device components able to measure physiological signals and either provide them as the raw input to the digital controller or in the form of a time series of measurements as an input to a signal processing or machine learning algorithm to produce some unified variable, an index to be used by the digital controller. In case the physiological signal is directly used by the controller, the variable may be affected by external environmental or physiological disturbances resulting in short-term failure in physiological signal monitoring, which can be referred to as statistical error. Without the ability to recognize and correctly treat these failures, the PCLC device may enter into a potentially hazardous state. When clinical staff are included in the loop, they may recognize and ignore these sensing anomalies. However, in a closed-loop controller configuration, these failures will be accepted by the controller and, therefore, may affect the therapy being delivered.

The theory of faults/anomalies detection in dynamic systems is vast (Willsky, 1976; Chandola et al., 2009). On the other hand, statistical sensor failures can be seen as medical alarms, while the detection of alarms in medical environments has also received a lot of attention (Burgos et al., 2010; Jiang et al., 2010; Saria et al., 2010; Ghorbani and Bogdan, 2013). The most common detectors in clinical environments are based on sensor threshold alarms when a single physiological value is monitored. Once the threshold of this sensor value is surpassed, an alarm is generated. Such an approach is subject to a high rate of false alarms. On the other hand, if, in certain situations, alarms are not generated, it can lead to multiple adverse events, including death (Bach et al., 2018). Recently, a novel approach for alarm detection based on a parameter-invariant design was proposed (Weimer et al., 2015) and applied to the detection of clinical pulmonary shunts in infants (Ivanov et al., 2015).

In case the physiological signal is first processed by an intermediate algorithm, the algorithm may take care of statistical errors, though the level of reliability, robustness, and trustworthiness of such algorithm plays a crucial role. A wrongly developed algorithm can introduce a systematic error to the final variable, which may not be noticed even by medical personnel, for example in the case of DoA index computation (An et al., 2017).

All three examples have their own way of building a sensor model. In the ICD example, the sensing model is represented by a set of EGM signatures and therefore resembles more of the population PK model of the anesthesia case. However, the “timing model instances" and further integration of prior statistics help to enrich this average model with inter-patient variability. Such modeling is essential for testing and evaluating discrimination algorithms, however, it will not allow to model and test cases of statistical sensor failures.

In the anesthesia case, in addition to inter-individual variability of physiological parameters used to generate a virtual cohort, it also provides the statistical measurement noise model in the form of intra-individual variability. It models only statistical measurement imprecision, however, the separation of the sensor and cohort generation model allows the introduction of occasional sensor failures outside of noise statistical distribution and thus evaluating system fault tolerance.

The strongest argument for using simulation of a representative cohort of patients to exhaustively test a closed-loop algorithm instead of performing animal testing is that the closed-loop algorithm is based on a predictive model developed using parameters of human beings, and therefore testing it on animals cannot be considered conclusive. This is true for all devices where decisions are taken on predictive models developed on human statistics, such as, for example, medication infusion pumps that are using human PK models.

### 4.2. Closed-loop controller

Basing the controller model solely on sensor measurements is not fully reliable, since measurements will be affected by noise and breakdowns. Moreover, an individual patient is subject to intra- and inter-individual variability. This, in turn, affects the physiological response, which requires that the controller performs over a wide range of uncertain conditions and therefore may lead to instability if the controller design is not sufficient for the expected range of physiological conditions. Moreover, the physiological response may come with certain delays, periods (depending on the sensing technologies), measurement errors, or external disturbances that will certainly add instability to controller performance.

Therefore, it is advisable to develop a closed-loop algorithm incorporating predictive physiological models and sensor measurements, such as Model Predictive Control (MPC) approaches. This way, in cases when MPC techniques are used to implement digital controllers, population physiological models are an essential derivative of the general physiological model.

### 4.3. Actuator

At first glance, the task of actuator modeling looks simple. However, here we must address such questions as limitations of the actuator imposed by:

- *Human physiology*: for example, the infusion rate of an IV drug delivery device must be limited to prevent harm to the patient's veins.
- *Limitations of the device* itself, such as the limitation of delivery speed adaptation or limited supply of resources, e.g., the limited volume of a syringe.
- *Clinical settings*, including human factor considerations and the user interface.

### 4.4. System components interoperability

PCLC devices that combine sensors, actuators, and controllers for patient treatment may have issues arising from interoperability among these system components, as well as their interaction with the environment. It is therefore important to provide a detailed specification of each system component, including their interaction model with the neighboring components in order to allow the detection of potential system failures. To fully address this question, one must refer to multiple already existing methodologies for system component-based design, such as formalization of system-level specification (Bauer et al., 2012) and the theory of contracts (Benveniste et al., 2018).

## 5. Conclusion and future work

Ensuring the safety, efficacy, and robustness of PCLC is not the only challenge that can be addressed by synthetic biomedical data generation and their application in ISCT. Multiple biomedical research domains would benefit from ISCT, which requires new model development. Generalization of the ISCT approach is also possible and can be seen as the ultimate goal of the ISCT efforts, however, a critical mass of relevant ISCT case studies still needs to be reached.

The experience of the pharma domain has shown that the development of new drugs has resulted in more strict regulatory requirements that, in the end, have become affordable only to big industries. As a result, Novartis is one of the biggest representatives of the pharma industry in the market, while small pharmacists had to stop the research and development of their proprietary drugs because they could not afford the competition.

Can the early adoption of physiological models and the performance of ISCT at all stages of the medical device life cycle allow for lowering the cost of design, development, and registration of novel PCLC medical devices and thus prevent the monopolization of the market? That remains to be seen. However, it can only be seen if an attempt toward the development of ISCT based on the generation of big synthetic biomedical data is made.

## Author contributions

The author confirms being the sole contributor of this work and has approved it for publication.

## Footnotes

^1^ https://www.fortunebusinessinsights.com/industry-reports/medical-devices-market-100085

^2^ https://www.trade.gov/country-commercial-guides/switzerland-healthcare

^3^ https://avicenna-alliance.com/

^4^ https://www.fda.gov/medical-devices/digital-health-center-excellence

^5^ https://www.chuv.ch/en/bdsc/

^6^ www.fda.gov/

^7^ www.ema.europa.eu

^8^ www.swissmedic.ch/

^9^ https://www.arcomed.com/infusion-pumps/anesthesia/
